# Infant feeding practice and associated factors among HIV positive mothers in Debre Markos Referral Hospital East Gojam zone, North West Ethiopia

**DOI:** 10.11604/pamj.2016.24.300.8528

**Published:** 2016-08-05

**Authors:** Elias Bekele Wakwoya, Tatek Abate Zewudie, Kahsay Zenebe Gebresilasie

**Affiliations:** 1 School of Nursing and Midwifery, College of Health and Medical science, Haramaya University, Harar, Ethiopia; 2 Ethiopian Midwives association, Addis Ababa, Ethiopia; 3 Department of Midwifery, College of Medicine and Health Sciences, University of Gondar, Gondar, Ethiopia

**Keywords:** HIV, infant, feeding, factors, practice

## Abstract

**Introduction:**

The dilemma posed between lifesaving benefit and risk of transmission through breast feeding complicates infant feedings among communities grossly affected by HIV/AIDS. According to the world health organization’s guideline which was revised in 2010, exclusive breast feeding and exclusive replacement feeding are the recommended infant feeding practices for HIV positive mothers. The aim of this study was to assess infant feeding practice and associated factors among HIV positive mothers in Debre Markos Referral Hospital, North West Ethiopia.

**Methods:**

An institutional based cross sectional study was conducted from May to September 2013. A Randomly selected 260 HIV positive mothers were included. The data were collected by using a pretested and structured questionnaire. Bivariate and multivariate analysis were performed to check association and to control confounders.

**Results:**

From a total of 260 HIV positive mothers, 85.8% of them were feeding their children based on the recommended feeding way of infant feeding practice with the remaining percentage 14.2% were practicing mixed feeding. In multivariate analysis mothers attending high school and above AOR = 5.3 [95% CI = 1.25-22.1], having antenatal care follow up AOR = 5.5 [95% CI = 1.5-20.16], being on anti-retro viral therapy AOR = 6.5 [95% CI = 1.88-22.51] and disclosure of HIV status AOR = 7.1 [95% CI = 1.26-39.76] were found to be independently associated with infant feeding practice.

**Conclusion:**

This study revealed that large proportion of HIV positive mothers had followed the recommended infant feeding practice and significantly high number of mothers had practiced mixed feeding. Educating mothers, increasing ANC utilization, counseling mothers to start ART, encouraging and supporting mothers to disclose their HIV status were recommended.

## Introduction

Globally 34.0 million people were living with HIV by the end of 2011. In the same year 3.3 million children were living with HIV and more than 90% of them were living in sub-Saharan Africa. In Ethiopia the point estimate of the total HIV population among children aged 0-14 years in 2012 were 168,598 [1]. In sub Saharan Africa, mother to child transmission (MTCT) of HIV is responsible for about 90% of infection in children, and about half of these infection thought have been acquired through breast feeding. The availability of ART alone does not solve the problem of HIV transmission through breastfeeding which is responsible for as much as 5-20% [2]. The dilemma posed between life saving benefit and risk of transmission through breastfeeding complicate infant feedings in a communities affected by HIV/AIDS. The main challenge is how to improve, through optimal feeding the nutritional status, growth and healthy development and thus survival of infants and young children in the given circumstances in the middle of HIV/AIDS pandemics [3]. The World Health Organizations guideline on HIV and infant feeding which was revised in 2010 recommends that in order to reduce the risk of the baby becoming infected, mothers or their infants are advised to take a course of antiretroviral drugs through the breastfeeding period.

Mothers are also advised to exclusively breastfeed their infants for 6 months meanwhile introduces other food substances while continuing breastfeed up to a year. The guideline also lists special conditions needed to safely formula feed [3]. Some studies revealed that the risk of mother to child transmission is lower among mothers who exclusively breastfed when compared to those who practiced mixed feeding. Evidences also indicates that exclusive replacement feeding (ERF) is associated with low risk of post natal HIV transmission and high mortality when compared to breast feeding [4–6]. The type and duration of recommended infant feeding practices is one of the critical decisions that should be made to maximize prevention benefits for the child. This is also the part of strategies of PMTCT in a plan to create the future HIV free generation. However there was no information regarding the infant feeding practice and associated factors of the HIV positive mothers in Debre Markos referral hospital which supposed to serve over 5 million peoples. It also provides evidence based information to policy makers and stake holders to exert maximum effort in improving recommended feeding practice for HIV positive mothers.

## Methods

A cross sectional study was conducted in Debre Markos referral hospital, North West Ethiopia, from May to September, 2014. Debre Markos town is located in east Gojam zone of Amhara regional state, North West Ethiopia 300 kilometers far from the capital Addis Ababa. The total population of the town was 62,497 out of this 52.1% were females and 48.9% were males. The source populations were all HIV positive mothers who had an infant or last child less than or equal to 2 years of age attending PMTCT and services and or on ARV treatment in Debre Markos referral hospital. Study population were all HIV positive mothers who had an infant or last child less than or equal to 2 years of age attending PMTCT services and or on ARV treatment in Debre Markos referral hospital and were volunteer to participate in the study. The sample size was determined by using single population proportion formula based on the following assumption; proportion of RFP 89.5% taken form study done in Gondar Ethiopia, 95% confidence level (Z =1.96) and 5% margin of error and the final sample size was 260. Systematic random sampling technique was employed to select the study participants and the intervals were found to be 2 and the starting point was selected randomly. Therefore all mothers who came at every second interval were recruited until the final 260 HIV positive mothers were obtained. The data was collected by face to face interview by using structured and pretested questionnaire. To ensure the quality of data, training was given for two days for data collectors by the principal investigators. The collected data were entered into Epi Info version 3.5.1 and then exported into SPSS version 20.0 software package for analysis. After cleaning the data for internal consistency descriptive statistics like frequencies and percentages were calculated. Odds ratios and 95% confidence interval were computed to determine the presence and the degree of association between dependent and independent variables. P value less than 0.05 was considered to decide statistical significance. More over multivariate logistic regression was employed to control confounders. Ethical clearance was obtained from ethical review committee of University of Gondar College of medicine and health science. Supporting letter was obtained from university of Gondar to Debre Markos referral hospital. In addition informed consent was obtained from each study participants prior to interview.

## Results

### Socio-demographic characteristic of the participants

A total of 260 HIV positive mothers in Debre Markos referral hospital were included in the study making a response rate of 100%. The mean age of mothers and their children were 29.9 (SD ± 4.47) years and 9.15 (SD ± 6.93) months respectively. Majority, 214(82.3%) of mothers were married, 255(98.1%) of mothers were Amhara in ethnicity and 258(99.2%) were orthodox Christian by religion. More than one third of the mothers 92(35.4%) had no formal education and 65 (30.4%) of husbands has completed elementary school. Regarding the occupational status of mothers 97(37.3%) were house wives followed by daily laborers and employees 70(26.9%) and 35(13.5%) respectively. The mean monthly income of mothers was 913.25ETB (Table 1).

**Table 1:** Sociodemographic characteristics of HIV positive mothers in Debre Markos Referral Hospital, 2014

Variable	Number	Percent %
**Age of mothers**		
18-24	30	11.5
25-29	120	46.2
30-34	74	28.5
+35	36	13.8
**Age group of the children**		
≤ 5 months	114	43.8
6-11 months	57	21.9
12-24 months	89	34.2
**Marital status**		
Married	214	82.3
Divorced	18	6.2
Single	21	8.1
Widowed	7	2.7
**Ethnicity**		
Amahara	255	98.1
Others (Oromo, Agawu, Tigre)	5	1.9
**Religion**		
Orthodox	258	99.2
Muslim	2	0.8
**Mothers educational level**		
Unable to read and write	92	35.4
Read and write	24	9.2
Grade 1-8	64	24.6
Grade 9-12	39	15.0
12 completed and above	41	15.8
**Occupation of mothers**		
Housewife	97	37.3
Employee	52	20.0
Daily laborer	70	26.9
Merchant	27	10.4
Others (farmer, student)	14	5.4
**Husbands educational level**		
Unable to read and write	27	12.6
Read and write	15	7.0
Grade 1-8	63	29.4
Grade 9-12	65	30.4
12 completed and above	44	20.6
**Income**		
<=500	109	41.9
501-1000	83	31.9
<=1001	68	26.2

### Obstetrics and ARV prophylaxis history and disclosure status of HIV positive mothers

From the total 260 HIV positive mothers 213(81.9%) were having antenatal care follow up. Majority of mothers 226(86.9%) gave birth at government health institutions and the remaining 34(13.1%) deliveries were at home. From those who delivered at health institution most of them were having SVD 221(85%) followed by cesarean section 24(9.2%). One hundred eighty four (70.8%) of mothers have started ART. The majority of infants 224(86.2%) get the ART prophylaxis after delivery. The greatest proportion of mothers 231(88.8%) had disclosed their HIV status (Table 2).

**Table 2:** Obstetric and ARV prophylaxis history and disclosure status of HIV positive mothers in Debre Markos referral hospital, 2014

Variables	Frequency	Percent %
ANC follow up		
Yes	213	81.9
No	47	18.1
Place of delivery		
Governmental HI	226	86.9
Home	34	13.1
Type of delivery		
SVD	221	85.0
SVD with episiotomy	13	5.0
Cesarean section	24	9.2
Instrumental delivery	2	0.8
Ever started ART		
Yes	184	70.8
No	76	29.2
ART prophylaxis given during pregnancy		
Yes	30	39.5
No	46	60.5
ART prophylaxis given to the child		
Yes	224	86.2
No	36	13.8
Disclosure of HIV status		
Yes	227	87.3
No	33	12.7

### Knowledge and infant feeding practice of HIV positive mothers

**Figure 1:**
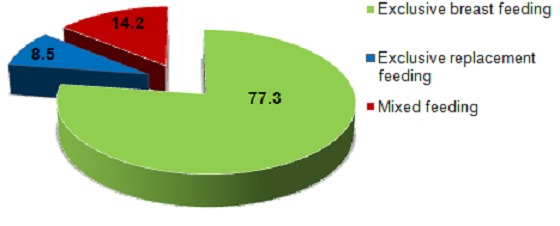
Percentage of infant feeding practice of HIV positive mothers in Debre Markos Referral Hospital Western Ethiopia, September 2014

**Figure 2:**
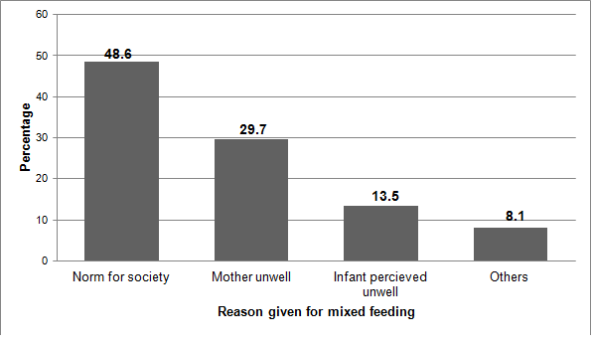
Reasons for mixed feeding of HIV positive mothers in Debre Markos Referral Hospital Western Ethiopia, September 2014

Large proportion, 239 (91.9%) respondents had adequate knowledge towards infant feeding. From a total of 260 HIV, positive mothers, 238 (91.5%) have ever breast feed their babies (Table 3). Two hundred one (77.3%) of respondents practiced exclusive breast feeding whereas 37(14.2%) and 22(8.5%) practiced mixed feeding and exclusive replacement feeding respectively (Figure 1). Regarding the initiation of breastfeeding the majority of mothers 162(62.3%) breastfed their babies within the first hour after delivery Norm of society and 18(48.7%) and mother unwell 11(28.9%), were the major reasons cited by respondents who practiced mixed feeding (Figure 2). The frequently given food for infants in addition to breast milk was cow milk 34(91.9%). Only two respondents had ever practiced wet nursing (Table 4). Twenty two (8.5%) of this mothers had ever expressed their breast milk, but only 16(72.7%) gave the expressed to their children and none of them have treated it with heat. Among the mothers who practiced ERF 22(8.5%), most of them used commercial infant formula and all of respondents have used cup and spoon to provide these foods (Table 4).

**Table 3:** Knowledge of HIV positive mothers on infant feeding in Debre Markos referral hospital, September 2014

Variable	Number	Percent %
Ever heard about infant feeding (N=260)		
Yes	252	96.9
No	8	3.1
Source of information (N=260)		
Neighbors	16	6.2
Husband	11	4.2
Health professionals	184	70.8
Mass media	65	25.0
Knowledge of mothers on IFO (N=260)		
Adequate	239	91.9
Inadequate	21	8.1

**Table 4:** Feeding practice of HIV positive mothers in Debre Markos referral hospital, September 2014

Variables	Frequency	Percent %
Ever breast feed (N=260)		
Yes	238	91.5
No	22	8.5
Time of first initiation of breast milk		
First 1 hour	162	68.1
First 8 hours	40	16.8
After 8 hours	36	15.1
Infant received any food or fluid before the first breast milk		
Yes	17	7.14
No	221	92.86
What foods or fluids given before 6 months		
Cow milk	34	91.9
Porridge/cereal based foods	19	51.3
Others (adult food, formula milk)	8	21.6
Anyone ever breast feed your child		
Yes	2	0.8
No	258	99.2
Ever expressed breast milk		
Yes	22	8.5
No	237	91.5
Ever gave the expressed milk		
Yes	16	72.7
No	6	27.3
Reason for expressed breast milk		
To separate from infant	8	36.4
Infant unable to suck	8	36.4
Due to breast pain	6	27.2
Kind of food used for replacement feeding		
Commercial infant formula	14	63.6
Home prepared formula	4	18.2
Both alternatively	4	18.2
Complementary food started		
Yes	150	57.7
No	110	42.3

### Feeding practice of HIV positive mothers

**Figure 3:**
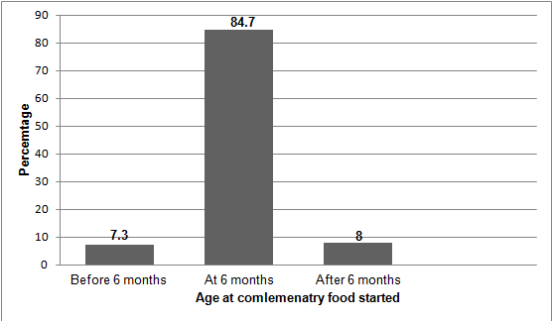
Age at complementary food started for infants of HIV positive mothers in Debre Markos Referral Hospital Western Ethiopia, September 2014

From a total of 260 HIV positive mothers, the greatest proportion of mothers 238(91.5%) ever breast feed their babies. 201(77.3%) of respondents practiced exclusive breast feeding whereas 37(14.2%) and 22(8.5%) practiced mixed feeding and exclusive replacement feeding respectively (Figure 3). The majority of mothers 162(62.3%) breast feed their babies within the first hour after delivery. Seventeen (6.5%) of mothers give foods to their babies before the first breast milk and among these 13 (76.5%) of them give butter. Norm of society 18(48.65%) and mother unwell 11(28.9%), were the major reasons cited by respondents who practiced mixed feeding. The commonly given food for the infants in addition to breast milk was Cow milk 34(91.9%). Only two (0.8%) respondents had ever practiced wet nursing (Table 4). From a total of 260 mothers 22(6.2%) of HIV positive mothers had ever expressed their breast milk; of whom only 16(72.7%) gave the expressed breast milk to their children and none of them have treated the expressed milk with heat before they gave to their babies. The major reasons were to separate from the infant and infant unable to suckle on breast feeding 8(36.36%). Half of the respondents used bottle 8(50%) where as the other half used cup 8(50%) (Table 4). Among the mothers who practiced ERF 22(8.5%), most of them 14(63.63%) used commercial infant formula and all of the respondents 22(100%) used cup and spoon to provide these foods (Table 4). Majority of the respondents 127(84.67%) started complementary food at the age of 6 months (Figure 1).

### Factors affecting infant feeding practices

In binary logistic regression analysis mother’s educational level COR = 3.65 [95% CI = 1.29-10.34], ANC follow up COR = 11.9 [95% CI = 1.96-8.22] and being on ART COR = 4.01 [95% CI = 1.96-8.22] were associated with feeding practice. Likewise place of delivery, disclosure of HIV status and knowledge on infant feeding COR =5.21 [95% CI = 2.32-11.72], COR = 19 [95% CI= 8.1-44.7] and COR = 3.5 [95% CI = 1.3-9.3] respectively were also associated with infant feeding practice. In multivariate analysis mother’s educational status, ANC follow up, being on ART and disclosure of HIV status were found to be independently associated (p-value <0.05) with infant feeding practice. Those mothers who had educational level of high school and above were 5.25 times more likely to follow recommended way of infant feeding practice than those who were unable to read and write AOR = 5.25[95% CI = 1.25-22.1]. Mothers who had ANC follow up were 5.5 times more likely to follow RFP compared to those who had no ANC follow up AOR = 5.5[95% CI= 1.5-20.16]. Mothers who have been on ART were 6.5 times more likely to follow RFP than those who were not on ART; AOR = 6.5[95% CI = 1.88-22.51]. The odd of practicing RFP was 7.1 times more likely in those who disclose their HIV status with their spouse. AOR = 7.1[ 95% CI = 1.26-39.76] (Table 5).

**Table 5:** Bivariate and multivariate logistic regression analysis showing relation between recommended feeding practice and selected variable of HIV positive mothers In Debre Markos referral hospital, September 2014

Variables	Feeding practice		COR [95% CI]	AOR [95% CI]
	RFP (%)	NRFP (%)		
**Mothers educational level**				
Unable to read and write	74(78.9)	18(13.1)	1	1
Read and write	16(66.7)	8(33.3)	0.49[.18-1.31]	0.32[.07-1.47]
Grade 1-8	58(90.6)	6(9.4)	2.35[.88-6.3]	3.67[.85-15.92]
High school and above	75(93.8)	5(6.2)	3.65[1.29-10.34]	5.25[1.25-22.1]*
**Husband educational level**				
Unable to read and write	21(77.8)	6(22.2)	0.65[0.23-1.8]	
Read and write	11(73.3)	4(26.7)	0.51[.15-1.78]	
Grade 1-8	58(92.1)	5(7.9)	2.14[0.75-6.13]	
High school and above	92(84.4)	17(15.6)	1	
**Income**				
≤ 500	92(84.4)	17(15.6)	1	
501-1000	68(81.9)	15(18.1)	0.84[.39-1.79]	
>1000	63(97.1)	5(2.9)	2.33[.82-6.64]	
**ANC follow up**				
Yes	198(93.0)	15(7.0)	11.6[5.34-25.27]	5.5[1.5-20.16]*
No	25(53.2)	22(46.8)	1	1
**On ART**				
Yes	168(91.3)	16(8.7)	4.01[1.96-8.22]	6.5[1.88-22.51]*
No	55(72.4)	21(27.6)	1	1
**Place of delivery**				
Government HI	202(89.4)	24(10.6)	5.21[2.32-11.72]	
Home	21(61.8)	13(38.2)	1	
**Disclosure of HIV status**				
Yes	210(92.5)	17(7.5)	19[8.1-44.7]	7.1[1.26-39.76]*
No	13(60.6)	20(39.4)	1	1
**Knowledge of mothers on infant feeding**				
Adequate	209(87.4)	30(12.6)	3.5[1.3-9.3]	
Inadequate	14(66.7)	7(33.3)	1	

## Discussion

This study has tried to assess infant feeding practice and associated factors among HIV positive mothers in Debre Markos Referral hospital, North West Ethiopia. In this study the proportion of respondents who practiced EBF 2.1(77.1%) is consistent with the finding reported from Gondar (83.8%); however it was higher than the findings of Addis Ababa, India and South Africa which was (30.6%), (47.7%) and (27%) respectively [7–10]. The possible reason for this difference is participants of these comparative studies were more of relied on replacement feeding. This study revealed that the proportion of respondents who practiced ERF were 22(8.5%). This finding was in line with the study conducted in eastern Uganda (8.5%) and Gondar (5.7%) [10, 11]. But lower than what was reported from India (51.3%), South Africa (50%) and Addis Ababa (46.8%) [7–9]. These discrepancies might be due to difference in culture of feeding habit, study time, economic potential, health policy and strategies of intervention. Mothers who choose to breast feed their infants should never practice mixed feeding as this may increase the risk of HIV transmission and illness or death from diarrhea and other illnesses. The rate of mixed feeding among participants in the present study (14.2%) is in line with study done in Kenya (14%), Addis Ababa (15.3%), and Gondar (10.5%) [7, 10, 12]. However it is comparatively higher than the study conducted in Cameroon (4.3%) [13]. This difference might be due to all participants included in the comparative study area were those who previously seen at the prenatal period and counseled on infant feeding options, this probably helped them to follow the recommended feeding options and few proportion of respondents who practiced mixed feeding compared to this study. On the other hand the proportion of respondents who practiced MF in the present study was comparatively less than what was reported from eastern Uganda (51%) and Abidjan, Cote d’ivoire (39%) [14, 15]. The discrepancy can be explained as; in the comparative studies the proportion of mixed feeding was computed among mothers with infants less than 6 months of age whereas in the present study mothers who had a child above 6 months of age were also included. In the present study the commonest reason raised by respondents who practiced mixed feeding was norm of society (48.6%) while neighbor’s advice (40%) was a major reason reported from Addis Ababa [7]. This highlighted that there was a deep rooted norm that made mothers to practice mixed feeding in both study areas. This study also revealed that, among mothers who ever breast feed, 162(68%) of mothers initiated breast milk within one hour of delivery. This finding was comparatively lower than the study done in Gondar (82.3%) [10]. The discrepancy might be due to the difference in characteristics of study population and the study area.

Ethiopian ministry of health Guideline on PMTCT recommends introduction of complementary food at six months of age. In the present study the majority of respondents 127(84.7%) have started complementary feeding at 6 months of age. However in Lusaka, Zambia more than half of participants (52%) started complementary feeding at 4 months [16]. This could be due to the difference in sample size and study period. In this study maternal educational status, ANC follow up, being on ART and disclosure of HIV status to spouse were significantly and independently associated with feeding practice. This study revealed that mothers who had educational level of high school and above were 5.3 times more likely to follow recommended way of infant feeding practice than those who were unable to read and write. This finding is consistent with study done in Southern Ghana [17]. This might be due to the fact that; as mothers are educated their decision making power increases and this helps them to practice the recommended IFO irrespective of the pressure from partner, family and society as compared to those mothers who were uneducated. Mothers who had ANC follow up were 5.5 times more likely to follow the recommended feeding option than those who had no ANC follow up. This finding is consistent with what was reported from India, where numbers of ANC visits were positively associated with ideal infant feeding options [18]. This might be due to; mothers who had ANC follow up had a high chance of obtaining counseling on recommended feeding options as compared to mothers who had no ANC follow up. Even though being on ART was not indicated as factor influencing infant feeding options in any of the comparative study, it was significantly associated with RFP in the present study. Mothers who have been on ART were 6.5 times more likely to follow RFP than those who are not on ART. The possible reason could be due to; health professionals follow the infant feeding practice of mothers during their visit to ART. This study also showed that mothers who disclosed their HIV status with their spouses were 7 times more likely to follow recommended feeding options than those who did not disclosed their HIV status. This finding is consistent with study done in Tanzania and Northwest Ethiopia [10, 19]. This could be explained as; mothers who have disclosed their HIV status with their spouses could have open discussion on what to feed their baby these could further increase the involvement of partners in infant feeding decision and encourage mothers to follow RFP.

## Conclusion

The study revealed that large proportion of HIV positive mothers followed the recommended infant feeding practice and significantly high number of mothers had practiced mixed feeding. The proportion of mothers who followed the RFP in this study was almost similar with that of other studies conducted in Ethiopia. Maternal educational status, ANC follow up, being on ART and disclosure of HIV status were significantly associated with recommended way of infant feeding practice.

### What is known about this topic

- Since there is no study done on this topic in Debre Markos Hospital nothing is known about this topic.

### What this study adds

- The prevalence of mixed feeding among HIV positive mothers is still not few;
- Number of antenatal care follow up is associated with feeding practice;
- The knowledge of mothers on recommended feeding practice is high.
